# Sonographic sunray appearance of Ewing’s sarcoma of skull

**DOI:** 10.11604/pamj.2021.39.152.30216

**Published:** 2021-06-28

**Authors:** Rohan Kumar Singh, Gaurav Vedprakash Mishra

**Affiliations:** 1 Department of Radiodiagnosis, Jawaharlal Nehru Medical College, Datta Meghe Institute of Medical Sciences, Sawangi (Meghe), Wardha, India,

**Keywords:** Ewing’s sarcoma, sunray appearance, ultrasound, pediatric bone tumor

## Image in medicine

A 3-year-old male child with his mother visited our hospital after being referred from primary health care centre with chief complains of rapidly growing swelling over left temporal region for 2 months. On physical examination, the swelling measured 3.1 x 3.3cm and firm in consistency, non-compressible, non-tender with no discoloration of overlying skin. The child was afebrile with no associated cervical lymphadenopathy. Systemic and neurological status was normal. An ultrasound of the local site showed well defined heterogeneously hypoechoic lesion with linear hyper-echogenicity. Increased vascularity was noted on color Doppler, the findings consistent with typical sunray appearance. A diagnosis of Primary Ewing's sarcoma was made. Ewing's sarcoma is a common pediatric bone tumor. Sunray appearance is a typical feature which results from spiculated type of periosteal reaction seen on ultrasound, an important non-radiation diagnostic tool. Treatment is planned based on location and extent of tumor spread.

**Figure 1:**
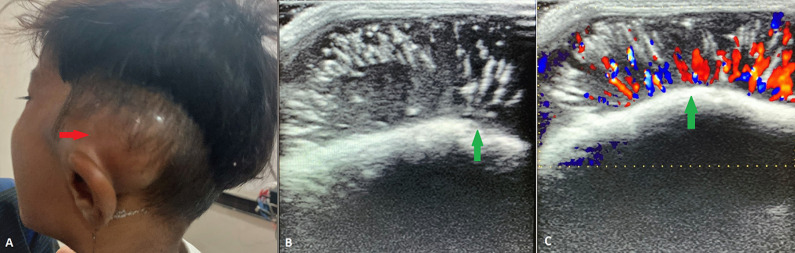
A) clinical image of swelling in post-auricular region above the temporal bone (red arrow); B) ultrasound image showing a hypoechoic mass lesion with sunray appearance (green arrow); C) color Doppler image showing increased vascularity in a linear branching pattern of sunray appearance (green arrow)

